# Mapping Patterns of Parental Burnout Along Psychological Resources and Parenting Styles

**DOI:** 10.3390/bs16071051

**Published:** 2026-06-24

**Authors:** Patrik M. Bogdán, Katalin Varga, Szandra Katona, Kristóf Gróf, Annamária Pakai

**Affiliations:** 1 Doctoral School of Health Sciences, Faculty of Health Sciences, University of Pécs, H-7621 Pécs, Hungary; kristof.grof@etk.pte.hu; 2 Department of Affective Psychology, Faculty of Education and Psychology, Eötvös Loránd University, H-1064 Budapest, Hungary; varga.katalin@ppk.elte.hu; 3 Faculty of Health Sciences, Institute of Basics of Health Sciences, Midwifery and Health Visiting, University of Pécs, H-7621 Pécs, Hungary; kat.szandi@gmail.com; 4 Faculty of Health Sciences, Institute of Emergency Care, Pedagogy of Health and Nursing Sciences, University of Pécs, H-7621 Pécs, Hungary; annamaria.pakai@etk.pte.hu

**Keywords:** parental burnout, parenting styles, resilience, emotional exhaustion, resource depletion, parenting identity, stress and coping, family functioning

## Abstract

Background: Parental burnout results from chronic stress related to the parental role and reflects a persistent imbalance between parenting demands and available psychological resources, negatively affecting parental well-being and parent–child relationships. This study examined the associations between parental burnout, parenting attitudes, and psychological resilience within the parental adaptation of the job demands–resources model, with particular attention to the potential mediating role of parenting styles in the relationship between resilience and parental burnout, while controlling for sociodemographic factors. Methods: A cross-sectional design was applied with 447 Hungarian parents who completed an anonymous online questionnaire including the Parental Burnout Assessment, the Parenting Styles and Dimensions Questionnaire, and the 10-item Connor–Davidson Resilience Scale. Data were analyzed using nonparametric correlations, group comparisons, multiple linear regression models with bootstrap estimation, and mediation analyses. Results: Resilience showed negative associations with all dimensions of parental burnout. Authoritarian and permissive parenting styles were positively associated with burnout, whereas authoritative parenting style showed negative associations. In multivariate analyses, authoritative parenting attitudes and fulfillment of the ideal parental role emerged as protective factors, while authoritarian parenting style functioned as a significant risk factor. Mediation analyses further indicated that the association between resilience and parental burnout may partly operate through parenting styles, particularly across the dimensions of emotional exhaustion, contrast, and emotional distancing. Conclusions: Parental burnout appears to be a dynamic psychological process shaped by the interaction of internal resources and parenting functioning, underscoring the importance of resource-oriented approaches in prevention and intervention.

## 1. Introduction

                      “*There is no such thing as a perfect parent. So just be a real one.*”

Sue Atkins

Parental burnout (PB) is a state of psychological, emotional, and physical exhaustion resulting from prolonged strain and represents a response to chronic stress associated with the parental role (Roskam et al., 2021). PB is a multidimensional construct comprising several interrelated yet distinct components. According to the literature, four main dimensions can be identified. Emotional exhaustion in the parental role, emotional distancing from one’s child, a sense of inadequacy in the parental role, and a feeling of being fed up with parenting (Roskam et al., 2021). Together, these dimensions capture the complexity of parental burnout, while also being associated with different psychological mechanisms and outcomes. In recent years, increasing attention has been devoted to understanding and mapping this phenomenon, which shows a growing prevalence (Piotrowski et al., 2023). Parenthood has become increasingly demanding as a consequence of dynamic social changes over recent decades (Nomaguchi & Milkie, 2020). These changes can be identified at several levels. For example, the increasing emphasis on career building has led to a postponement of childbearing, with families deciding to have children at progressively later ages (Zabak et al., 2023). An additional challenge arises from idealized images of parenting originating from multiple sources, which in many cases appear in society as norms to be achieved or as minimum expectations (Lin et al., 2022).

In today’s accelerated world, it has become increasingly common for parents to experience substantial work-related demands, and multiple job holding is also becoming more frequent. Ongoing economic difficulties likewise represent a significant factor within the parental role, as childrearing is associated with considerable financial burdens (Spitzer et al., 2022). Difficulties related to childrearing may manifest across several domains. These include everyday parenting activities required for establishing basic normative frameworks, as well as parental tasks related to guidance, support, and control, which become particularly salient during puberty and adolescence (Kobak et al., 2017). In addition, expectations and challenges associated with children’s education may place a substantial burden on parents.

The level of chronic stress may be further increased when a child is affected by a mental or physical condition (e.g., Down syndrome, special educational needs (SEN), ADHD, neurodivergence) (Zonta et al., 2024). With regard to parental burnout (PB), an additional risk factor is the presence of a current or previous mental disorder in either parent, such as generalized anxiety disorder or depression (Yakupova & Suarez, 2023). Parents with low levels of social support, as well as those characterized by excessive perfectionism in relation to childrearing, can also be considered particularly vulnerable (Mikolajczak & Roskam, 2018).

These risk factors have been empirically linked to the development of parental burnout (PB) in international research. Parental burnout does not only affect parents’ psychological well-being, but may also have significant implications for children’s development and overall family functioning. From a family systems perspective, parental functioning is closely linked to children’s emotional security, behavioural regulation, and general well-being. Previous research suggests that higher levels of parental burnout are associated with less adaptive parenting patterns, which, in turn, may be linked to less favourable child adjustment and developmental outcomes.

The adverse family and emotional environment associated with parental burnout may also have longer-term implications for children’s development (Kalkan et al., 2022). Some studies indicate that children raised in such environments more frequently exhibit adjustment difficulties, behavioural problems, or challenges in emotion regulation (Goodman et al., 2010). Furthermore, emotionally less supportive parenting may be associated with later interpersonal difficulties, as well as increased vulnerability to risk-taking behaviours, including substance use (Whitesell et al., 2013; Wall & Kohl, 2007). Recent studies have also highlighted that parental burnout is associated with parenting quality, child adjustment, and broader relational processes within the family (Carone et al., 2026). Taken together, these considerations underscore the importance of examining parental burnout not only at the individual level, but also within a broader developmental and societal context.

To better understand these mechanisms, it is particularly important to examine higher-order psychological dimensions that may play a key role in the development and maintenance of parental burnout. One such higher-level dimension is parenting attitude or style (parenting styles; PS), the theoretical foundation of which was established by Diana Baumrind. Parenting style refers to the constellation of behaviors and attitudes that are applied, consciously or unconsciously, during childrearing (Baumrind, 1967).

Over the long term, parenting style shapes the child’s perceptions of the self, others, and the world, and influences social and emotional functioning as well as personality development (Awiszus et al., 2022). It plays a significant role in the expression of parental influence, in the child’s everyday well-being, and in the quality of the parent–child relationship (Darling & Steinberg, 1993). Specific dimensions of parenting attitudes (PS), such as authoritarian, authoritative, and permissive styles, have a defining role in the quality of parent–child interactions and, indirectly, in the mental well-being of both parents and children (Delvecchio et al., 2020).

Authoritarian parents are characterized by low responsiveness to the child’s behavior and needs and by low levels of emotional involvement. They compel the child to follow strict rules, suppress the child’s striving for autonomy, and expect unquestionable and unconditional obedience. Parent–child communication is often one-sided, in which the child’s expressed needs and emotions are not met with adequate or supportive responses. Permissive parents typically maintain a warm and emotionally close relationship with the child and respond to the child’s needs with high levels of responsiveness; however, expectations regarding the child’s behavior are low, and boundaries are broadly defined. Authoritative parents apply an optimal balance of the characteristics described above during childrearing. They are characterized by deep emotional involvement and respond sensitively to the child’s needs.

Within the parent–child relationship, communication is balanced; the child is treated as an autonomous individual and supported in autonomy striving, while this occurs within clearly defined and dynamic boundaries accompanied by continuous monitoring. The systems of discipline and reward are consistent and appropriately adapted to the situation (Kuppens & Ceulemans, 2018).

Another key explanatory factor of parental burnout (PB) is resilience, defined as the capacity for effective coping with stress. Although research on resilience has a long history and its mediating role in parental burnout has been supported by several studies, its association with parenting attitudes has received considerably less emphasis in the international literature to date (Ren et al., 2024).

Parental burnout, parenting attitudes, and resilience can be integrated into a well-established and relevant psychological framework, the theoretical foundation of which is provided by the job demands–resources model (JD–R) developed by Demerouti and Bakker, and later adapted to the context of parental burnout by Isabelle Roskam and Moïra Mikolajczak. According to their formulation, parental burnout can be interpreted, based on the simplest adaptation of the JD–R model, as a condition that emerges when “parental demands are high while parental resources are low” (Demerouti et al., 2001; Mikolajczak & Roskam, 2018, A theoretical framework for parental burnout, para. 2).

Within this framework, resilience can clearly be interpreted as an internal psychological resource. In contrast, parenting attitude cannot be understood exclusively as a resource, but rather as a mode of functioning that simultaneously reflects the parent’s current psychological resources and level of strain. Accordingly, parenting style may be interpreted both as a protective factor and as a behavioral consequence of parental burnout. The demand side of the parental role is represented by the mental and physical burdens associated with parenting, as well as by sociodemographic and life-context factors, such as the child’s health status, family structure, social support, or economic conditions. The present study primarily focuses on resource-related factors, with particular emphasis on resilience and parenting attitudes, while capturing certain aspects of parental demands through sociodemographic and background variables. Parental burnout, parenting functioning, and resilience thus form a dynamic system of interacting factors, in which individual components may simultaneously be interpreted as risk factors and outcomes.

Previous research has extensively examined parental burnout, particularly with regard to its sociodemographic and contextual determinants. In addition, a growing body of evidence highlights the role of higher-order psychological constructs, such as resilience. However, relatively few studies have examined the combined effects of higher-order psychological factors—such as resilience and parenting styles—on parental burnout within a multivariate framework. Furthermore, it remains insufficiently understood how these factors are interrelated and through which mechanisms they influence burnout.

Investigating these relationships may contribute to a deeper understanding of how psychological resources are reflected in parenting functioning and how they relate to the different dimensions of parental burnout, particularly in terms of a more differentiated interpretation of its various aspects.

The present study seeks to address this gap by examining resilience and parenting styles within an integrated model grounded in the Job Demands–Resources (JD–R) framework. To the best of the authors’ knowledge, this is the first study conducted in Hungary to simultaneously analyse parental burnout, parenting attitudes, and psychological resources within a multivariate model. This approach allows for the examination of the independent effects of these factors and contributes to a more nuanced understanding of the psychological mechanisms underlying parental burnout.

### 1.1. Aim of the Study

The aim of the present study is to examine specific dimensions of parental burnout within a multivariate analytical framework, as well as their associations with parenting attitudes and resilience. The study seeks to provide a comprehensive overview of the patterns of parental burnout that are linked to different forms of parenting functioning, and to clarify the contexts in which these associations can be interpreted.

In addition, the research addresses the role of parents’ sociodemographic and socioeconomic characteristics, as well as the ways in which certain child-related characteristics are associated with patterns across the dimensions of parental burnout. The objective of the analysis is not limited to the description of co-occurring relationships, but also aims at a deeper understanding of the associations between parental burnout and parenting attitudes, which may contribute to the identification of risk and protective factors and to the grounding of prevention- and intervention-oriented practices.

#### Hypothesis

The development of parental burnout is the result of the combined influence of multiple factors, in which the balance between parental demands and available psychological resources plays a key role. Higher-level psychological factors, such as resilience and parenting styles, may influence parental functioning and the development of burnout not only directly but also in relation to each other. In addition, certain life circumstances—such as raising a child with special educational needs or a diagnosis of ADHD—may contribute to the risk of burnout through increased demands placed on the parent.

Due to the cross-sectional design of the study, causal inferences regarding the direction of associations cannot be drawn; therefore, the hypotheses are formulated in a non-causal and partly exploratory manner.

**H1.**  *We assume that resilience is associated with parenting styles, such that higher levels of resilience may be related to more adaptive parenting patterns (i.e., lower levels of permissive and authoritarian styles and higher levels of authoritative style); however, the direction of this relationship cannot be clearly determined due to the cross-sectional design.*

**H2.**  *We assume that authoritarian and permissive parenting styles may show a positive association with parental burnout; however, these relationships are not necessarily unidirectional, as parental burnout may also influence parenting practices.*

**H3.**  *We assume that higher levels of parental burnout are observed among parents raising children with special educational needs or diagnosed with ADHD, which may be associated with increased parental demands.*

**H4.**  *We assume that parenting styles may play a mediating role in the relationship between resilience and parental burnout, as psychological resources may exert their effects through parental functioning.*

## 2. Materials and Methods

A descriptive, cross-sectional, quantitative study design was applied in the present research. The study was conducted with the approval of the Scientific and Research Ethics Committee. Ethical approval number: BM/23020-3/2024.

### 2.1. Sampling Method

Using a non-probability convenience sampling procedure, the sample included men and women aged 18 years or older who were living in Hungary, spoke Hungarian, and were raising at least one child, with the youngest child being at least 3 years of age. Parents who had a diagnosed mental disorder at the time of the study (e.g., generalized anxiety disorder or depression) were excluded from the sample. Parents were also excluded if any of their children had a severe physical or mental disability, as such conditions may substantially increase the psychological burden placed on the parent. The exclusion criterion regarding severe physical or mental disabilities was based on parental self-report. Consistent with the approved ethical protocol and the principle of data minimization, participants were not asked to provide detailed diagnostic, functional, or medical information. Instead, eligibility was determined using a screening question regarding the presence of severe physical or mental disability in the child, accompanied by illustrative examples (e.g., Down syndrome, intellectual disability).

In contrast, certain conditions such as ADHD or special educational needs were included in the study, as they may be relevant to parental burnout through increased parenting-related demands, while still being interpretable within general patterns of parental functioning.

These criteria were applied to ensure that the study focused on mechanisms of parental burnout as observed in the general population, while minimizing the potential confounding effects of extreme caregiving burden.

No strict upper age limit was defined during sampling, as it has become increasingly common for parents to have children at later stages of life, and thus parents over the age of 50 may also be raising young children. Accordingly, the flexible treatment of the upper age limit contributed to better reflecting current social realities and to increasing the social validity of the study.

The decision to include only parents with a youngest child aged at least 3 years was based on methodological considerations. First, some of the instruments used in the study assess parent–child interaction patterns that are not yet stable or meaningfully interpretable in children under the age of three. Second, early parenthood may involve substantial overlap between parental burnout, postpartum-related symptoms, sleep deprivation, and normative early parenting strain, which may complicate construct differentiation and measurement validity during early developmental stages. 

### 2.2. Sample Size Estimation

The minimum required sample size was estimated using the G*Power software, version 3.1.9.7, assuming small-to-moderate effect sizes (correlation: *r* = 0.20; two-group difference: *d* = 0.30), with an alpha level of 0.05 and a statistical power of 0.80. Based on these calculations, an approximate sample size of *N* ≈ 190–200 was sufficient for correlational analyses (Spearman’s rank correlation), whereas a total sample of N ≈ 340–360 was required for two-group comparisons (Mann–Whitney U test). Accordingly, the minimum required sample size was determined by the procedure demanding the largest sample, resulting in an estimated lower threshold of approximately 350 participants (Faul et al., 2009). For multivariate regression analyses, commonly cited guidelines from the literature were taken into account, according to which reliable estimation of parameters requires at least 10–15 cases per predictor. The planned sample size met these criteria; therefore, the application of the regression models can be considered statistically justified.

### 2.3. Data Collection Procedure

Data collection was conducted using an online, anonymous questionnaire. Completion required approximately 10–15 min, and participation was entirely voluntary. The questionnaire did not collect any personally identifiable information (such as names, addresses, email addresses, or telephone numbers), and data analysis was performed exclusively on aggregated data. Distribution of the questionnaire was supported by professionals involved in family support and therapeutic services, and the call for participation was also shared on several relevant social media platforms.

The questionnaire package included the Parental Burnout Assessment (PBA) scale and the Parenting Styles and Dimensions Questionnaire (PSDQ), in addition to sociodemographic variables. As part of the sociodemographic section, an additional variable was assessed to capture the subjective evaluation of the fulfilment of the ideal parental role. Participants responded to a single-item question (“To what extent do you feel you have fulfilled your ideal parental role?”), rated on a 0–10 scale (0 = not at all; 10 = completely), with higher scores indicating greater perceived fulfilment of the ideal parental role. Physical punishment was assessed using a single dichotomous self-report item asking participants whether they had ever used physical punishment toward their child during childrearing (e.g., slapping the child’s hand or spanking) (“Have you ever used physical punishment toward your child, such as slapping the hand or spanking?” Yes/No). The frequency and severity of physical punishment were not assessed.

The Parental Burnout Assessment (PBA) is a measurement instrument developed by Belgian researchers, the Hungarian-language adaptation and psychometric validation of which were conducted by Hamvai and colleagues, with the results published in 2022 (Hamvai et al., 2022). Based on the national validation, the reliability indices of the instrument proved to be excellent, and similarly to the original version, the Hungarian version also demonstrated a four-factor structure representing the dimensions of parental burnout. The 23-item scale showed high internal consistency across its subscales, with Cronbach’s alpha values ranging between 0.84 and 0.93, while the reliability of the total scale was also outstanding. Items of the questionnaire—for example, “I do not enjoy being with my children”—are rated on a seven-point Likert scale according to the frequency with which the given statement characterizes the respondent (0 = never; 6 = every day). Higher scores indicate a greater presence of parental burnout within the given dimension. The instrument does not include any reverse-scored items.

The Parenting Styles and Dimensions Questionnaire (PSDQ) was developed by Robinson and colleagues in 2001. The Hungarian-language validation was conducted by Hadházi and colleagues in 2021 (Hadházi et al., 2021). The 32-item questionnaire assesses three parenting styles: authoritarian, authoritative (also referred to as directive or democratic), and permissive. Responses are given on a five-point Likert scale indicating frequency, measuring how often parents apply certain behaviors toward their child (e.g., “When my child behaves well, I praise him or her”). The authoritarian and authoritative subscales demonstrated high Cronbach’s alpha values, whereas the reliability of the permissive subscale was lower (α = 0.635). This finding is consistent with the international literature, which indicates that for subscales with a low number of items, Cronbach’s alpha often underestimates true reliability. The questionnaire does not include any reverse-scored items.

Resilience was assessed using the 10-item, unidimensional instrument developed by Connor and Davidson (CD-RISC-10), which represents a reduced version of the original 25-item, three-dimensional scale. The Hungarian-language adaptation was conducted by Járai and colleagues in 2015. As a result of the validation process, the resilience questionnaire showed correlations of appropriate direction and magnitude with the instruments used for comparison. Cronbach’s alpha was 0.85, which is comparable to the reliability reported for the original questionnaire. Items of the scale are rated on a Likert-type scale according to how often the statement applies to the respondent (e.g., “I work to achieve my goals”), with response options ranging from 0 = not true at all, 1 = rarely true, 2 = sometimes true, 3 = often true, to 4 = almost always true. The questionnaire does not include any reverse-scored items (Járai et al., 2015).

### 2.4. Software Used for Data Analysis

Microsoft Excel 2016 was used for the organization and preparation of raw data, while IBM SPSS Statistics 25 was applied for statistical analyses. Descriptive statistical procedures were employed to present the basic characteristics of the sample, including the calculation of means and standard deviations, minimum and maximum values, as well as absolute and relative frequencies. Based on the examination of variable distributions, the assumptions of normality were not met; therefore, nonparametric statistical procedures were applied to explore bivariate associations, including Spearman’s rank correlation and the Mann–Whitney U test. To examine multivariate relationships, multiple linear regression analyses were conducted, allowing for the assessment of how individual predictor variables were associated with the examined outcome variables while simultaneously controlling for background variables. To further examine the relationship between resilience and parental burnout, a mediation analysis was conducted using the PROCESS macro (Model 4; Hayes, 2022). In the model, resilience was entered as the independent variable, parenting styles as parallel mediators, and the dimensions of parental burnout as outcome variables. Indirect effects were estimated using 5000 bootstrap resamples with 95% confidence intervals (Efron & Tibshirani, 1994). Results are reported with 95% confidence intervals, and the threshold for statistical significance was set at *p* < 0.05. Cases with missing data were not included in the analyses.

## 3. Results

A total of 548 participants were initially recruited for the study, including 416 mothers and 132 fathers. Following the application of the exclusion criteria, 101 participants were excluded, resulting in a final sample size of 447 participants. In the final sample, 27.1% were men (*n* = 121) and 72.9% were women (*n* = 326). The mean age of participants was 39.15 years (SD = 6.5). The youngest participant was 21 years old, while the oldest was 59 years old. A detailed overview of the sociodemographic characteristics of the sample is presented in Table 1.

### 3.1. Distributional Properties of the Variables and Internal Reliability of the Measures

During the study, normality testing of the study variables was conducted, and the internal reliability of the applied questionnaires was examined using Cronbach’s alpha and McDonald’s omega statistics, in order to confirm the psychometric reliability and applicability of the measurement instruments. The results of the normality tests and the reliability indices are presented in Table 2 and Table 3.

The distribution of age was slightly right-skewed (skewness = 0.442) and characterized by kurtosis close to normality (kurtosis = −0.190). The Shapiro–Wilk test indicated a significant deviation from normality (*p* < 0.001). The mean score of the Connor–Davidson Resilience Scale was 2.694 (SD = 0.663), indicating a moderately high level of resilience. Skewness was slightly negative (−0.473), suggesting a higher frequency of higher resilience values, while kurtosis (−0.306) indicated a distribution close to normal with slight platykurtosis. According to the significant result of the Shapiro–Wilk test (*p* ≤ 0.001), the distribution deviated from normality.

The mean scores of all subscales of the Parental Burnout Assessment (PBA) fell within the low range (Emotional Distancing: M = 1.596; Contrast: M = 1.053; Saturation: M = 0.987; Emotional Withdrawal: M = 0.807), indicating that parental burnout was generally present at a low level in the sample. All subscales showed positive skewness (1.053–1.867), suggesting that lower burnout scores were more frequent, whereas higher values occurred less often. Kurtosis was higher for several subscales (e.g., 3.588 for the PBA Emotional Distancing subscale), indicating more peaked distributions. The Shapiro–Wilk test was significant for all subscales (*p* ≤ 0.001), meaning that the distributions cannot be considered normal.

Among the subscales of the Parenting Styles and Dimensions Questionnaire (PSDQ), the mean score of the permissive parenting style was 2.307, the authoritarian style was 1.633, and the authoritative style was 4.089, the latter showing the highest value. This indicates that the authoritative parenting attitude was the most characteristic in the sample. The permissive and authoritarian subscales showed slightly positive skewness (0.401 and 0.952, respectively), whereas the skewness of the authoritative attitude was negative (−0.721), indicating a higher frequency of higher values. Kurtosis values were within a range close to normality. The normality test was significant for all of these subscales (*p* ≤ 0.001), a pattern that is commonly observed in the case of Likert-type scales.

All subscales of the Parental Burnout Assessment as well as the Connor–Davidson 10-item Resilience Scale demonstrated high reliability according to both Cronbach’s alpha and McDonald’s omega indices. The authoritarian (ω = 0.797; α = 0.784) and authoritative (ω = 0.859; α = 0.855) subscales of the Parenting Styles and Dimensions Questionnaire also indicated high internal reliability, whereas the permissive subscale (ω = 0.580; α = 0.570) showed lower reliability. When interpreting the lower reliability values, it should be taken into account that this subscale consists of only five items, which in itself may contribute to more moderate Cronbach’s alpha and McDonald’s omega coefficients.

### 3.2. Examination of Associations Between the Variables

Due to deviations of the variable distributions from normality and the presence of background variables measured on an ordinal scale, Spearman’s rank correlation was applied to analyze associations between variables. Group differences were examined using nonparametric tests. For comparisons between two groups, the Mann–Whitney U test was used. The correlation matrix presented in Table 4 includes Spearman’s correlation coefficients for all examined variables. 

Based on the results of the Spearman rank correlation analysis, age showed a significant, weak negative association with social isolation due to parenting (ρ = −0.200, *p* < 0.01), emotional exhaustion (ρ = −0.173, *p* < 0.01), the contrast dimension (ρ = −0.102, *p* < 0.05), and the saturation dimension (ρ = −0.167, *p* < 0.01). This indicates that older parents report lower levels of certain aspects of parental burnout as well as lower levels of perceived social isolation.

Educational attainment showed a significant positive association with emotional exhaustion (ρ = 0.219, *p* < 0.01), the saturation dimension (ρ = 0.196, *p* < 0.01), and emotional distancing (ρ = 0.158, *p* < 0.01).

Fulfillment of the ideal parental role showed a significant negative association with social isolation due to parenting (ρ = −0.246, *p* < 0.01), and a significant, moderate positive association with resilience (ρ = 0.386, *p* < 0.01). In addition, it demonstrated significant moderate-to-strong negative associations with all subscales of parental burnout: emotional exhaustion (ρ = −0.436, *p* < 0.01), the contrast dimension (ρ = −0.583, *p* < 0.01), the saturation dimension (ρ = −0.458, *p* < 0.01), and emotional distancing (ρ = −0.344, *p* < 0.01).

Social isolation due to parenting showed a significant negative association with resilience (ρ = −0.231, *p* < 0.01), while exhibiting significant positive associations with emotional exhaustion (ρ = 0.446, *p* < 0.01), contrast (ρ = 0.432, *p* < 0.01), saturation (ρ = 0.414, *p* < 0.01), and emotional distancing subscales (ρ = 0.263, *p* < 0.01).

Resilience showed significant negative associations with all subscales of parental burnout: emotional exhaustion (ρ = −0.325, *p* < 0.01), contrast (ρ = −0.401, *p* < 0.01), saturation (ρ = −0.305, *p* < 0.01), and emotional distancing (ρ = −0.272, *p* < 0.01).

Consistent and significant associations were observed between parenting styles and the subscales of parental burnout. Emotional exhaustion was positively associated with the permissive parenting style (ρ = 0.288, *p* < 0.01) and the authoritarian style (ρ = 0.332, *p* < 0.01), while showing a negative association with the authoritative parenting style (ρ = −0.268, *p* < 0.01).

The contrast dimension was positively correlated with the permissive (ρ = 0.275, *p* < 0.01) and authoritarian parenting styles (ρ = 0.412, *p* < 0.01), and negatively correlated with the authoritative style (ρ = −0.324, *p* < 0.01). The saturation dimension showed significant positive associations with the permissive (ρ = 0.208, *p* < 0.01) and authoritarian styles (ρ = 0.329, *p* < 0.01), and a negative association with the authoritative style (ρ = −0.287, *p* < 0.01).

Emotional distancing was positively associated with the permissive (ρ = 0.260, *p* < 0.01) and authoritarian parenting styles (ρ = 0.344, *p* < 0.01), and negatively associated with the authoritative style (ρ = −0.343, *p* < 0.01).

Fulfilment of the ideal parental role was also significantly associated with parenting styles. It showed negative associations with the permissive (ρ = −0.313, *p* < 0.01) and authoritarian styles (ρ = −0.371, *p* < 0.01), and a positive association with the authoritative style (ρ = 0.300, *p* < 0.01).

Social isolation due to parenting showed a weak but significant negative association with the authoritative parenting style (ρ = −0.114, *p* < 0.05).

Resilience was significantly and negatively associated with the permissive (ρ = −0.242, *p* < 0.01) and authoritarian parenting styles (ρ = −0.243, *p* < 0.01), while showing a positive association with the authoritative parenting style (ρ = 0.341, *p* < 0.01)

#### Results of Two-Group Comparisons (Mann–Whitney U Test)

Gender differences were examined using the Mann–Whitney U test. In all analyses, the sample sizes were as follows: men *n* = 121 and women n = 326 (*N* = 447). Effect size was expressed using the “*r*” value, calculated as the ratio of the absolute value of the *Z* statistic associated with the Mann–Whitney test to the square root of the total sample size.

For resilience, a significant difference was observed between men and women (*U* = 16,774, Z = −2.433, *p* = 0.015), with men showing a higher mean rank (Mean Rank = 248.37) than women (Mean Rank = 214.95), indicating that men had significantly higher levels of resilience (*r* = 0.115).

A significant gender difference was also observed on the emotional exhaustion subscale of parental burnout (*U* = 14,965, *Z* = −3.923, *p* < 0.001), with women showing a higher mean rank (Mean Rank = 238.60) than men (Mean Rank = 184.68), indicating higher levels of emotional exhaustion among women (*r* = 0.186).

Differences were likewise identified on the PBA contrast subscale (*U* = 14,690, *Z* = −4.172, *p* < 0.001): the mean rank of women (Mean Rank = 239.44) exceeded that of men (Mean Rank = 182.40), indicating a stronger experience of contrast with the parental role among women (*r* = 0.197). A similar pattern emerged for the saturation subscale (*U* = 14,790, *Z* = −4.101, *p* < 0.001), with women having a higher mean rank (Mean Rank = 239.13) than men (Mean Rank = 183.23) (*r* = 0.194).

No statistically significant gender differences were found on the emotional distancing subscale or across any of the three dimensions of parenting attitudes (permissive, authoritarian, authoritative) (*p* > 0.05).

With regard to marital status, 45 participants were single parents and 402 were living in a relationship (*N* = 447). Single parents demonstrated higher resilience scores than parents living in a relationship (*U* = 7023.5, *Z* = −2.463, *p* = 0.014), with single parents showing a higher mean rank (Mean Rank = 268.92) than parents living in a relationship (Mean Rank = 218.97). This indicates that single parents had significantly higher levels of resilience (r = 0.116).

Among parenting attitudes, a significant difference was found for the authoritarian parenting style (*U* = 6461, *Z* = −3.154, *p* = 0.002). Parents living in a relationship showed a higher mean rank (Mean Rank = 230.43) than single parents (Mean Rank = 166.58), indicating a stronger presence of the authoritarian parenting attitude among parents living in a relationship (*r* = 0.149).

The authoritative parenting style also differed between the two groups (*U* = 6636.5, *Z* = −2.934, *p* = 0.003). Single parents showed a higher mean rank (Mean Rank = 277.52) than parents living in a relationship (Mean Rank = 218.01), indicating a stronger presence of the authoritative parenting style among single parents (*r* = 0.139). No statistically significant differences were found between single parents and those living in a relationship with regard to the subscales of parental burnout or the permissive parenting style.

With respect to the availability of external support in childrearing, 114 participants reported no external assistance, whereas 333 participants indicated having external support (*N* = 447). Parents without external support reported higher emotional exhaustion scores (*U* = 15,865.5, *Z* = −2.619, *p* = 0.009). Parents who did not have external support in childrearing showed a higher mean rank (Mean Rank = 251.33) than those who received external support (Mean Rank = 214.64), indicating that the lack of external support is associated with higher levels of emotional exhaustion (*r* = 0.142).

Differences were also observed on the PBA contrast subscale (*U* = 16,581, *Z* = −2.028, *p* = 0.043). Parents without external support had a higher mean rank (Mean Rank = 245.05) than those with external support (Mean Rank = 216.79), suggesting a stronger experience of discrepancy between the ideal and the lived parental functioning in this group. The effect size was small in this case as well (*r* = 0.096).

On the emotional distancing subscale, a borderline significant difference was observed (*U* = 16,738, *Z* = −1.963, *p* = 0.050). Parents without external support showed a higher mean rank (Mean Rank = 243.68) than those receiving external support (Mean Rank = 217.26), indicating a greater degree of emotional distancing in the parent–child relationship (*r* = 0.093). No statistically significant differences were found for resilience, the saturation dimension of parental burnout, or parenting attitudes.

With regard to the use of physical punishment, 314 participants reported having used physical punishment during childrearing, while 128 participants indicated that they had not used such a parenting practice. For this variable, data were missing for five participants (*N* = 447; *n* = 442).

Parents who reported using physical punishment showed higher emotional exhaustion scores (*U* = 15,882.50; *Z* = −3.461; *p* = 0.001). Parents who reported using physical punishment showed a higher mean rank (Mean Rank = 234.92) than those who did not use physical punishment (Mean Rank = 188.58), indicating higher levels of emotional exhaustion in this group (*r* = 0.165).

A significant difference was also found for the contrast dimension (*U* = 15,702.50; *Z* = −3.629; *p* < 0.001). The mean rank of parents who reported using physical punishment (Mean Rank = 235.49) exceeded that of parents who did not report such practices (Mean Rank = 187.18), suggesting a stronger experience of contrast with the parental role among these parents (*r* = 0.173).

Higher saturation scores were likewise observed among parents reporting physical punishment (*U* = 16,682.50; *Z* = −2.827; *p* = 0.005). Parents who reported using physical punishment showed a higher mean rank (Mean Rank = 232.37) than those who did not use physical punishment (Mean Rank = 194.83), indicating a more pronounced sense of overload and saturation related to the parental role in this group (*r* = 0.134).

A significant difference was also observed for the emotional distancing dimension (*U* = 16,819.00; *Z* = −2.807; *p* = 0.005). The mean rank of parents who reported using physical punishment (Mean Rank = 231.94) was higher than that of parents who did not report such practices (Mean Rank = 195.90), indicating a stronger level of emotional distancing toward the child in this group (*r* = 0.134).

Group differences were additionally identified for the permissive parenting style (*U* = 14,673.00; *Z* = −4.478; *p* < 0.001). Parents who reported using physical punishment showed a higher mean rank (Mean Rank = 238.77) than those who did not use physical punishment (Mean Rank = 179.13), suggesting a stronger presence of permissive parenting behavior in this group (*r* = 0.213).

A particularly pronounced difference was observed for the authoritarian parenting style (*U* = 8793.00; *Z* = −9.307; *p* < 0.001). Parents who reported using physical punishment showed a substantially higher mean rank (Mean Rank = 257.50) than those who did not report such practices (Mean Rank = 133.20), indicating a very strong presence of authoritarian parenting attitudes in this group (*r* = 0.443).

The authoritative parenting style also showed differences between the groups (*U* = 17,412.50; *Z* = −2.206; *p* = 0.027). Parents who did not report using physical punishment had a higher mean rank (Mean Rank = 242.46) than those who reported such practices (Mean Rank = 212.95), suggesting that the authoritative parenting style is more characteristic of non-abusive parents (*r* = 0.105).

### 3.3. Multiple Linear Regression Analysis of Predictors of Parental Burnout

A multiple linear regression analyses were conducted to examine the combined effects of factors influencing specific dimensions of parental burnout. The aim of these models was to identify which psychological and background variables independently contributed to the explanation of the different aspects of parental burnout, while controlling for the effects of the remaining variables.

In the regression analyses, the subscales of the Parental Burnout Assessment (PBA)—emotional exhaustion, contrast, saturation, and emotional distancing—were entered sequentially as dependent variables. Independent variables included resilience, dimensions of parenting attitudes (permissive, authoritarian, and authoritative), as well as relevant sociodemographic and environmental background factors (gender, age, number of children, marital status, availability of external support in childcare, educational attainment, and income level). Dichotomous variables were entered into the models using dummy coding.

The assumptions underlying the regression models were examined prior to the analyses. Multicollinearity was assessed using tolerance values and variance inflation factors (VIF), while potentially influential observations exerting excessive impact on the models were identified using Cook’s distance. The VIF values of the predictor variables were close to 1, indicating the absence of multicollinearity.

To enhance the reliability of the regression coefficient estimates, a bootstrap procedure with 5000 resamples was applied, allowing for the calculation of robust standard errors and confidence intervals. This approach was particularly warranted given the non-normal distribution of several variables and the potential presence of outliers.

Missing data were handled using listwise deletion; that is, only cases with valid data on all variables included in a given model were retained for analysis. As a consequence, the sample size of the regression models was reduced relative to the original sample. Nevertheless, this approach ensured the statistical validity and comparability of the estimates, assuming that missingness occurred completely at random (Missing Completely at Random, MCAR) (*N* = 447; *n* = 432; missing = 15).

This analytical strategy allowed the dimensions of parental burnout to be examined not only in relation to individual variables but also within a complex multivariate framework. In doing so, it enabled the identification of factors that retained independent explanatory power even after accounting for the effects of the other variables included in the models. The summary of the multiple linear regression analyses is presented in Table 5.

The regression model predicting emotional exhaustion showed good explanatory capacity (Adjusted *R*^2^ = 0.291; *F* (15, 416) = 12.816; *p* < 0.001). Permissive parenting style emerged as a significant positive predictor (*B* = 0.256; 95% CI: 0.032–0.481; *p* < 0.05), indicating that higher levels of permissiveness were associated with increased emotional exhaustion. In contrast, the authoritative parenting style showed a significant negative association with emotional exhaustion (*B* = −0.357; 95% CI: −0.635 to −0.079; *p* < 0.05). Gender was also a significant predictor (*B* = 0.435; 95% CI: 0.186–0.684; *p* < 0.01), suggesting that women reported higher levels of emotional exhaustion than men. The presence of an adolescent child was significantly and negatively associated with emotional exhaustion (*B* = −0.468; 95% CI: −0.750 to −0.187; *p* < 0.01). Fulfillment of the ideal parental role likewise proved to be a significant negative predictor (*B* = −0.206; 95% CI: −0.273 to −0.139; *p* < 0.001), indicating that greater perceived realization of the ideal parental role was associated with lower emotional exhaustion. Finally, the availability of external support in childcare was also linked to reduced emotional exhaustion (*B* = −0.396; 95% CI: −0.651 to −0.141; *p* < 0.01).

The model predicting emotional distancing was statistically significant (Adjusted *R*^2^ = 0.239; *F* (15, 416) = 10.017; *p* < 0.001). Authoritarian parenting style emerged as a significant positive predictor (*B* = 0.386; 95% CI: 0.026–0.745; *p* < 0.05), whereas authoritative parenting style showed a significant negative association with emotional distancing (*B* = –0.569; 95% CI: –0.810 to –0.328; *p* < 0.001). Fulfillment of the ideal parental role again functioned as a protective factor (*B* = −0.119; 95% CI: −0.177 to −0.061; *p* < 0.001). Similarly, the availability of support in childcare was associated with lower levels of emotional distancing (*B* = −0.292; 95% CI: −0.512 to −0.071; *p* < 0.05).

The model predicting the contrast subscale demonstrated strong explanatory power (Adjusted *R*^2^ = 0.378; *F* (15, 416) = 18.429; *p* < 0.001). Authoritarian parenting style emerged as a strong positive predictor (*B* = 0.648; 95% CI: 0.290–1.006; *p* < 0.001). In contrast, authoritative parenting style was negatively associated with contrast (*B* = −0.304; 95% CI: −0.544 to −0.064; *p* < 0.05). The presence of an adolescent child also showed a significant negative association with contrast (*B* = −0.292; 95% CI: −0.535 to −0.049; *p* < 0.05). Fulfillment of the ideal parental role again proved to be a particularly strong protective factor (*B* = −0.260; 95% CI: −0.318 to −0.203; *p* < 0.001). The presence of special educational needs was likewise associated with lower levels of contrast (*B* = −0.379; 95% CI: −0.700 to −0.058; *p* < 0.05). Finally, the availability of external support in childcare also contributed to reduced contrast (*B* = −0.281; 95% CI: −0.501 to −0.061; *p* < 0.05).

The regression model predicting the saturation subscale was also statistically significant (Adjusted *R*^2^ = 0.259; *F* (15, 416) = 11.052; *p* < 0.001). Authoritarian parenting style emerged as a significant positive predictor (*B* = 0.593; 95% CI: 0.227–0.959; *p* < 0.01). Gender again showed a significant effect (*B* = 0.299; 95% CI: 0.079–0.519; *p* < 0.01), indicating higher levels of saturation among women in the parental role. The presence of an adolescent child was associated with lower levels of saturation (*B* = −0.368; 95% CI: −0.616 to −0.119; *p* < 0.01). Fulfillment of the ideal parental role functioned as a protective factor in this model as well (*B* = −0.182; 95% CI: −0.241 to −0.123; *p* < 0.001). The presence of special educational needs (*B* = −0.369; 95% CI: −0.697 to −0.040; *p* < 0.05) and ADHD (*B* = 0.417; 95% CI: 0.059–0.775; *p* < 0.05) also emerged as significant predictors, with special educational needs showing a mitigating effect, whereas ADHD was associated with increased saturation. Finally, the availability of external support in childcare was linked to lower levels of saturation (*B* = −0.240; 95% CI: −0.465 to −0.015; *p* < 0.05), while monthly income showed a positive but weak association with saturation (*B* = 0.264; 95% CI: 0.004–0.524; *p* < 0.05).

### 3.4. Mediation Analysis of the Relationship Between Resilience and Parental Burnout

To further examine the relationship between resilience and parental burnout, a mediation analysis was conducted using the PROCESS macro (Model 4; Hayes, 2022). In the model, resilience was entered as the independent variable, parenting styles (permissive, authoritarian, and authoritative) as parallel mediators, and the dimensions of parental burnout as outcome variables. Indirect effects were estimated using 5000 bootstrap samples with 95% confidence intervals.

For the emotional exhaustion dimension, the total indirect effect of resilience on parental burnout was significant (Effect = −0.2494, 95% CI [−0.3643, −0.1488]), indicating that parenting styles mediate the relationship between resilience and burnout. All three parenting styles (permissive, authoritarian, and authoritative) showed significant indirect effects. The direct effect of resilience remained significant (Effect = −0.3110, *p* = 0.001), indicating partial mediation.

A similar pattern was observed for the contrast dimension. The total indirect effect was significant (Effect = −0.2545, 95% CI [−0.3619, −0.1559]), and all three parenting styles showed significant mediation effects, as none of the confidence intervals included zero. The direct effect of resilience also remained significant (Effect = −0.3306, *p* < 0.001), indicating partial mediation.

For the saturation (fed-up) dimension, the total indirect effect was also significant (Effect = −0.1912, 95% CI [−0.2911, −0.1014]). However, when examining the individual mediators, only the authoritarian parenting style showed a significant indirect effect, as its confidence interval did not include zero, whereas the confidence intervals for the permissive and authoritative styles included zero. The direct effect of resilience remained significant (Effect = −0.2112, *p* = 0.0119), again indicating partial mediation.

Finally, for the emotional distancing dimension, the total indirect effect was significant (Effect = −0.2612, 95% CI [−0.3739, −0.1640]). All three parenting styles showed significant indirect effects, as none of the confidence intervals included zero. The direct effect of resilience remained significant (Effect = −0.1571, *p* = 0.0439), indicating partial mediation. A summary of the indirect effects identified in the mediation analyses is presented in Table 6.

## 4. Discussion

The aim of the present study was to explore the associations between parental burnout, parenting styles, and resilience among Hungarian parents. Overall, our findings suggest that parental burnout cannot be interpreted solely as an individual psychological phenomenon, but rather emerges as a complex process involving psychological resources, parenting functioning, and social-environmental factors. This interpretative framework is consistent with Mikolajczak and Roskam’s “balance between risk factors and resources” model (Mikolajczak & Roskam, 2018).

Based on the main findings, authoritarian and permissive parenting styles, as well as social isolation, were associated with higher levels of parental burnout, whereas authoritative parenting, resilience, and a stronger sense of fulfilling the ideal parental role were linked to more favorable patterns. Overall, these findings are consistent with a substantial part of the hypotheses, although some results require a more nuanced interpretation.

Social isolation showed positive associations with all parental burnout subscales and a negative association with resilience (Zhao et al., 2023), supporting models emphasizing the stress-buffering role of social support (Cohen & Wills, 1985). According to Hobfoll’s Conservation of Resources theory, the absence of social relationships may increase the risk of resource loss (Hobfoll, 1989, 1998), although in the present study this theoretical framework primarily served an interpretative function.

The role of resilience demonstrated a particularly complex pattern. In the bivariate analyses, resilience showed negative associations with all burnout dimensions, consistent with approaches describing resilience as a protective factor (Connor & Davidson, 2003; Sorkkila & Aunola, 2021). This finding is in line with the expectations formulated in Hypothesis 1 at the correlational level; however, the study design does not allow for clear conclusions regarding the directionality of these relationships. In the multivariate regression models, resilience no longer emerged as an independent significant predictor, suggesting that its effect may not operate directly. This is also consistent with perspectives proposing that resilience may exert its influence through parenting functioning (Petrowski et al., 2014).

The mediation analyses further refined this interpretation, as the effect of resilience appeared partly through parenting styles. In the dimensions of emotional exhaustion, contrast, and emotional distancing, all three parenting styles demonstrated mediating effects, whereas in the case of saturation, only the mediating role of the authoritarian style was significant. Overall, these findings are consistent with Hypothesis 4, although the extent of mediation differed across dimensions. Importantly, due to the cross-sectional design, the directionality of the relationships cannot be clearly established. It is also possible that, in certain dimensions, the association between resilience and parental burnout operates to a greater extent through parenting styles.

Overall, the findings related to parenting styles are consistent with Hypothesis 2; however, results concerning the permissive parenting style should be interpreted cautiously due to the low reliability of the scale. The authoritarian parenting style showed positive associations with all burnout dimensions (Guo et al., 2024), whereas the authoritative style was consistently associated with more favorable patterns (Yaffe, 2023). The permissive parenting style also demonstrated positive associations, suggesting that lack of structure and inconsistent boundaries may increase parental uncertainty and emotional exhaustion, thereby contributing to cumulative stress development (Yang et al., 2025).

The fulfillment of the ideal parental role demonstrated a stable negative association with parental burnout dimensions across all models, which can also be interpreted in line with Hypothesis 2. This variable may represent an important indicator of parental identity, perceived competence, and psychological stability (Bandura, 1997), and may be associated with more adaptive parenting functioning and a more supportive developmental environment for the child (Albanese et al., 2019). Nevertheless, it should be considered that this construct was measured using a single item, which limits the interpretability of the findings.

In the case of physical abuse, the bivariate analyses indicated associations with higher levels of parental burnout (Mikolajczak et al., 2018), consistent with approaches linking increased psychological burden to maladaptive parenting responses (Herrenkohl et al., 2013). However, this effect did not remain significant in the regression models, suggesting that physical abuse may be better interpreted as an indicator of broader psychological and parenting dysfunction (Bahmani et al., 2022) rather than as an independent explanatory factor. It is also important to note that the present study did not assess emotional abuse or neglect, both of which may substantially affect child development (Norman et al., 2012; Tomoda et al., 2025).

Hypothesis 3 proposed that parents raising children with special educational needs or ADHD would report higher levels of parental burnout. However, our findings did not support this hypothesis. In the case of special educational needs, the direction of the association was contrary to expectations, with some dimensions being associated with lower burnout levels, whereas ADHD was associated with higher burnout only in the saturation dimension, suggesting a more nuanced and condition-specific pattern. The association observed in the saturation dimension may reflect the gradual depletion of parental resources (Nejatifar et al., 2025). One possible explanation for the lower burnout levels associated with special educational needs may be the presence of more structured professional support and the development of more realistic parental expectations (Çimen et al., 2023; Laila et al., 2025; Kellison et al., 2010), although the generalizability of these findings may be limited by contextual factors.

An interesting and partly unexpected finding was that single parents demonstrated higher resilience. Possible explanations may include self-selection, growth through coping experiences, or the development of greater autonomy; however, these interpretations should be approached cautiously due to the cross-sectional nature of the study. Our findings are also consistent with recent studies suggesting that parental burnout is embedded within broader family and relational processes (Carone et al., 2026; Tracchegiani et al., 2026).

Overall, our results suggest that parental burnout emerges through the combined influence of multiple factors and is closely associated with psychological resources, parenting functioning, and social factors.

Regarding practical implications, the most recent meta-analytic findings also indicate that psychological and educational interventions can effectively reduce parental burnout levels (Urbanowicz et al., 2025). The identified key components—psychoeducation, self-regulation, value-based approaches, and relational awareness—align closely with the patterns identified in the present study. Accordingly, our findings may contribute to the development of future interventions that simultaneously support parental resources and parenting functioning.

## 5. Conclusions

Our study was the first to explore the associations between parenting styles, resilience, and parental burnout in Hungary, and our findings were overall consistent with patterns described in the international and Central and Eastern European literature. Our results showed that authoritative parenting, higher resilience, and a stronger sense of fulfilling the ideal parental role were associated with more favorable psychological patterns, whereas authoritarian and permissive parenting styles, as well as social isolation, were associated with higher levels of parental burnout. The mediation analyses also suggested that the relationship between resilience and parental burnout may operate partly through parenting styles. These findings support the interpretation of parental burnout as a complex phenomenon closely related to psychological resources, parenting functioning, and social factors, in line with Mikolajczak and Roskam’s balance between risk and resource model (Mikolajczak & Roskam, 2018). Taken together, the findings suggest that parental burnout should not be understood solely as a consequence of parenting-related demands, but rather as a multidimensional phenomenon shaped by the interplay of psychological resources, parenting functioning, and social-contextual factors (Schrooyen et al., 2024).

Our findings may also have practical relevance, as they highlight the importance of supporting psychological resources, strengthening parental coping mechanisms, and promoting adaptive parenting functioning in the prevention and reduction of parental burnout. Based on the international literature, mindfulness-based interventions, emotion regulation techniques, and other forms of psychological support may effectively reduce parental burnout levels (Chaplin et al., 2021; Urbanowicz et al., 2025). The recent meta-analysis by Urbanowicz et al. (2025) also identified several shared components of effective interventions, including psychoeducation, self-regulation, values-based approaches, experiential practice, and relational awareness. These components closely align with the protective psychological and parenting-related factors identified in the present study, particularly resilience, adaptive parenting functioning, and supportive relational processes. In addition, our findings emphasize the importance of early recognition and preventive approaches within primary care and family support systems.

At the same time, some findings require cautious interpretation. In particular, the associations involving ADHD and special educational needs (SEN) were not entirely straightforward and may reflect multiple underlying mechanisms. The heterogeneity of developmental conditions, potential overlap between ADHD and SEN, differences in caregiving burden, and variation in access to support may all contribute to these patterns. Future studies should therefore employ more differentiated assessments of developmental conditions, caregiving burden, and support structures to clarify these relationships.

However, the cross-sectional nature of our study does not allow for clear conclusions regarding causality. Therefore, future research should prioritize longitudinal designs to better understand the directional and dynamic relationship between parental burnout and parenting styles, particularly among families experiencing increased psychological burden, such as parents raising children with special educational needs or ADHD.

## 6. Limitations

One of the main limitations of the present study is its cross-sectional design, which does not allow for causal inferences. Accordingly, the findings should be interpreted as associations rather than causal relationships. A further limitation is the use of non-probability sampling, which necessarily restricts the generalizability of the findings. As such, the sample is primarily representative of parents who were motivated to complete an online self-report questionnaire, and the results should therefore be generalized to the broader Hungarian parental population with caution.

An additional limitation of the present study is that only parents with a youngest child aged at least 3 years were included. While this decision was based on methodological considerations, it also implies that the study does not cover the early childhood period, which may represent a particularly sensitive phase in terms of parental burden. Consequently, the generalizability of the findings may be limited for parents of children under the age of three.

The relatively low number of fathers in the sample also limits the interpretability of the findings, particularly with regard to the examination of gender differences. Although this imbalance is consistent with general trends observed in similar research, it underscores the need for future studies to make more targeted efforts to involve fathers. From a methodological perspective, an additional limitation concerns the lower-than-desirable internal reliability of certain parenting attitude subscales, particularly the permissive parenting subscale (α = 0.570), which may reduce measurement precision within these dimensions and thereby attenuate the strength of the observed associations, potentially leading to an underestimation of the true effects. Accordingly, findings related to the permissive parenting subscale should be interpreted with caution and considered exploratory rather than confirmatory.

An additional limitation concerns the measurement of the “fulfilment of the ideal parental role” variable using a single-item indicator. Single-item measures may have limited reliability and validity and may not fully capture the complexity of the construct. Therefore, this limitation should be taken into account when interpreting the findings. A further methodological consideration relates to the use of multiple regression models with overlapping predictor variables, which may increase the risk of Type I error. As no formal correction procedures for multiple comparisons were applied, findings—particularly those with marginal significance levels—should be interpreted with caution. Physical punishment was assessed using a single dichotomous item asking whether the parent had ever used physical punishment toward their child. The frequency, severity, and temporal patterns of the behavior were not assessed, which limits the more detailed interpretability of this variable.

Parents with current mental disorders and families raising children with severe disabilities were excluded from the study. Although these exclusion criteria were applied to reduce substantial clinical heterogeneity, they may also have contributed to the formation of a relatively higher-functioning sample. Consequently, the generalizability of the findings may be limited with respect to populations at elevated risk for parental burnout. Furthermore, because detailed diagnostic and functional information regarding child developmental conditions was not collected, the boundaries between ADHD, special educational needs (SEN), and more severe developmental conditions could not be examined in greater detail. The heterogeneity of the SEN category, potential overlap between ADHD and SEN, and the exclusion of more severe cases may have influenced the observed associations. In addition, although differences in access to professional or educational support may partly explain some of the observed patterns, such support was not assessed in the present study; therefore, these interpretations should be considered speculative.

Finally, it is important to note that parenting attitude dimensions cannot be sharply separated either theoretically or empirically, as they show substantial overlap. Consequently, interpreting individual subscales in isolation may be misleading; instead, the findings should be understood as reflecting a complex and dynamic system of parenting functioning in which attitudes interact to shape the development of parental burnout.

Despite these limitations, the significance of the study is strengthened by the fact that, to our knowledge, no previous empirical research in Hungary has examined parental burnout, parenting attitudes, and resilience simultaneously within a multivariate statistical framework, using subscale-level analyses and accounting for relevant control variables. Accordingly, the present study fills an important gap in the literature by contributing to a deeper understanding of the complex psychological mechanisms underlying parental burnout and by providing a scientific basis for the development of evidence-informed prevention and intervention practices.

## Figures and Tables

**Table 1:** Sociodemographic Characteristics of the Participants.

Variable	*n* (*n* = 447)	%
Gender		
Male	121	27.1
Female	326	72.9
Marital Status	*n* (*n* = 447)	
Single	22	4.9
In a relationship or cohabiting	66	14.8
Married	336	75.2
Divorced	21	4.7
Widow	2	0.4
Type of residence	*n* (*n* = 447)	**%**
Village	59	13.2
Small town	54	12.1
City	180	40.3
County seat	95	21.3
Capital	59	13.2
Total monthly household income	*n* (*n* = 447)	**%**
100,000 Ft or below	1	0.2
101,000–200,000 Ft	3	0.7
201,000–300,000 Ft	9	2.0
301,000–400,000 Ft	25	5.6
401,000–500,000 Ft	47	10.5
Above 500,000 Ft	362	81.0
Level of education	*n* (*n* = 447)	**%**
Primary school (8 years)	6	1.3
Vocational school or “OKJ” qualification	65	14.5
Secondary school	127	28.4
College degree	117	26.2
University degree	126	28.2
PhD	6	1.3
Weekly working hours	*n* (*n* = 446) *	**%**
Unemployed	22	4.9
Currently on parental leave	90	20.1
Full-time, 40 h per week	193	43.2
More than 40 h per week	87	19.5
Less than 40 h per week	54	12.1

* Data on weekly working hours were missing for one participant (*n* = 446).

**Table 2:** Normality Assessment of the Study Variables.

	Age	Resilience	PBA Emot. Exh.	PBA Contrast	PBA Saturation	PBA Emot. Dist.	PSDQ Permissive	PSDQ Authoritarian	PSDQ Authoritative
Mean	39.15	2.694	1.596	1.053	0.987	0.807	2.307	1.633	4.089
Std. Deviation	6.507	0.663	1.341	1.244	1.168	1.127	0.576	0.363	0.465
Skewness	0.442	−0.473	1.053	1.640	1.765	1.867	0.401	0.952	−0.721
Kurtosis	−0.190	−0.306	0.498	2.166	3.164	3.588	0.115	1.955	0.778
Shapiro–Wilk	0.978	0.973	0.898	0.789	0.789	0.741	0.977	0.947	0.968
*p*-value of Shapiro–Wilk	<0.001	<0.001	<0.001	<0.001	<0.001	<0.001	<0.001	<0.001	<0.001
Minimum	21.00	0.800	0.000	0.000	0.000	0.000	1.000	1.000	2.200
Maximum	59.00	4.000	6.000	6.000	5.800	6.000	4.200	3.667	5.000

**Table 3:** Reliability Indices of the Questionnaires.

	Estimated McDonald-Omega + 95% CI	Estimated Cronbach-Alfa + 95% CI
PBA Subscales		
Emotional Exhaustion	0.930 (0.920–0.939)	0.927 (0.915–0.940)
Contrast	0.910 (0.897–0.923)	0.898 (0.878–0.918)
Saturation	0.912 (0.899–0.925)	0.898 (0.878–0.918)
Emotional Distancing	0.814 (0.784–0.844)	0.797 (0.745–0.849)
PSDQ Subscales		
Permissive	0.580 (0.520–0.640)	0.570 (0.503–0.638)
Authoritarian	0.797 (0.769–0.825)	0.784 (0.750–0.818)
Authoritative	0.859 (0.840–0.879)	0.855 (0.837–0.874)
Connor–Davidson Resilience Scale	0.868 (0.849–0.886)	0.861 (0.843–0.878)

**Table 4:** Correlation Matrix of the Study Variables.

	1.	2.	3.	4.	5.	6.	7.	8.	9.	10.	11.	12.
Age (1)	-	0.067	−0.006	−0.200 **	0.087	−0.173 **	−0.102 *	−0.167 **	−0.038	−0.073	0.013	0.057
Education (2)	0.067	-	−0.052	0.070	−0.017	0.219 **	0.090	−0.196 **	0.158 **	−0.056	−0.013	−0.067
Ideal parental role fulfillment (3)	−0.006	−0.052	-	−0.246 **	0.386 **	−0.436 **	−0.583 **	−0.458 **	−0.344 **	−0.313 **	−0.371 **	0.300 **
Social isolation due to parenting (4)	−0.200 **	00.070	−0.246 **	-	−0.231 **	0.446 **	0.432 **	0.414 **	0.263 **	00.054	00.082	−0.114 *
Resilience (5)	00.087	−00.017	0.386 **	−0.231 **	-	−0.325 **	−0.401 **	−0.305 **	−0.272 **	−0.242 **	−0.243 **	0.341 **
PBA_emotional exhaustion (6)	−0.173 **	0.219 **	−0.436 **	0.446 **	−0.325 **	-	0.734 **	0.818 **	0.657 **	0.288 **	0.332 **	−0.268 **
PBA_contrast (7)	−0.102 *	00.090	−0.583 **	0.432 **	−0.401 **	0.734 **	-	0.714 **	0.596 **	0.275 **	0.412 **	−0.324 **
PBA_Saturation (8)	−0.167 **	0.196 **	−0.458 **	0.414 **	−0.305 **	0.818 **	0.714 **	-	0.567 **	0.208 **	0.329 **	−0.287 **
PBA_emotionaldistancing (9)	−0.038	0.158 **	−0.344 **	0.263 **	−0.272 **	0.657 **	0.596 **	0.567 **	-	0.260 **	0.344 **	−0.343 **
PSDQ_permissive (10)	−0.073	−0.056	−0.313 **	0.054	−0.242 **	0.288 **	0.275 **	0.208 **	0.260 **	-	0.496 **	−0.226 **
PSDQ_authoritarian (11)	0.013	−0.013	−0.371 **	0.082	−0.243 **	0.332 **	0.412 **	0.329 **	0.344 **	0.496 **	-	−0.430 **
PSDQ_authoritative (12)	0.057	−0.067	0.300 **	−0.114 *	0.341 **	−0.268 **	−0.324 **	−0.287 **	−0.343 **	−0.226 **	−0.430 **	-

** Correlation is significant at the 0.01 level. * Correlation is significant at the 0.05 level.

**Table 5:** Summary of the Multiple Linear Regression Analyses.

Predictor	Emotional Exhaustion Unstandardized *B* (95% CI)	Emotional Distancing Unstandardized *B* (95% CI)	Contrast Unstandardized *B* (95% CI)	Saturation Unstandardized *B* (95% CI)
PSDQ Permissive	0.256 (0.032; 0.481) *	n.s	n.s	n.s
PSDQ Authoritarian	n.s	0.386 (0.026; 0.745) *	0.648 (0.290; 1.006) ***	0.593 (0.227; 0.959) **
PSDQ Authoritative	−0.357 (−0.635; −0.079) *	−0.569 (−0.810; −0.328) ***	−0.304 (−0.544; −0.064) *	n.s
Resilience	n.s	n.s	n.s	n.s
Sex	0.435 (0.186; 0.684) **	n.s	n.s	0.299 (0.079; 0.519) **
Age	n.s	n.s	n.s	n.s
Number of Children	n.s	n.s	n.s	n.s
Presence of an adolescent child	−0.468 (−0.750; −0.187) **	n.s	−0.292 (−0.535; −0.049) *	−0.368 (−0.616, −0.119) **
Fulfillment of the ideal parental role	−0.206 (−0.273; −0.139) ***	−0.119 (−0.177; −0.061) ***	−0.260 (−0.318; −0.203) ***	−0.182 (−0.241; −0.123) ***
Physical punishment	n.s	n.s	n.s	n.s
Special Education Needs	n.s	n.s	−0.379 (−0.700; −0.058) *	−0.369 (−0.697; −0.040) *
ADHD	n.s	n.s	n.s	0.417 (0.059; 0.775) *
Marital Status	n.s	n.s	n.s	n.s
External support in childcare	−0.396 (−0.651; −0.141) **	−0.292 (−0.512; −0.071) *	−0.281 (−0.501; −0.061) *	−0.240 (−0.465; −0.015) *
Income	n.s	n.s	n.s	0.264 (0.004; 0.524) *
Model fit (Adjusted R^2^, F (df1, df2), *p*-value)	0.291, F (15, 416) = 12.816; *p* < 0.001	0.239, F (15, 416) = 10.017; *p* < 0.001	0.378, F (15, 416) = 18.429; *p* < 0.001	0.259, F (15, 416) = 11.052; *p* < 0.001

* *p* < 0.05, ** *p* < 0.01, *** *p* < 0.001 (n.s = not significant).

**Table 6:** Indirect effects of resilience on the dimensions of parental burnout through parenting styles.

Burnout Dimension	Mediator	Indirect Effect	95% CI
**Emotional Exhaustion**	Permissive	−0.0798 *	[−0.1475; −0.0273]
	Authoritarian	−0.0757 *	[−0.1546; −0.0065]
	Authoritative	−0.0939 *	[−0.1824; −0.0171]
**Contrast**	Permissive	−0.0543 *	[−0.1160; −0.0042]
	Authoritarian	−0.1230 *	[−0.2055; −0.0554]
	Authoritative	−0.0773 *	[−0.1588; −0.0044]
**Saturation (Fed Up)**	Permissive	−0.0395	[−0.0932; 0.0027]
	Authoritarian	−0.0998 *	[−0.1741; −0.0364]
	Authoritative	−0.0519	[−0.1342; 0.0219]
**Emotional Distancing**	Permissive	−0.0572 *	[−0.1164; −0.0127]
	Authoritarian	−0.0656 *	[−0.1256; −0.0158]
	Authoritative	−0.1384 *	[−0.2264; −0.0627]

Note: Indirect effects were estimated using 5000 bootstrap samples. Values marked with an asterisk (*) indicate that the confidence interval does not include zero, and therefore the indirect effect is considered statistically significant.

## Data Availability

The dataset generated and analyzed during the current study is available from the corresponding author upon reasonable request. The data are not publicly available due to ethical and privacy considerations related to human participant research.
